# Medication Management Capacity Among Older Residents of Low-Income Apartment Buildings

**DOI:** 10.1093/geroni/igaa057.2747

**Published:** 2020-12-16

**Authors:** Amal Badawoud, Pamela Parsons, Juan Lu, Emily Peron, Teresa Salgado, Patricia Slattum

**Affiliations:** 1 Princess Nourah Bint Abdulrahman University, Riyadh, Saudi Arabia; 2 Virginia Commonwealth, Richmond, Virginia, United States; 3 Division of Epidemiology, Virginia Commonwealth University School of Medicine, richmond, Virginia, United States; 4 Virginia Commonwealth University School of Pharmacy, Richmond, Virginia, United States; 5 Virginia Commonwealth University, Richmond, Virginia, United States

## Abstract

MMC is an essential component of safe and independent living. This cross-sectional study was designed to assess MMC among low-income older persons residing in HUD-subsidized housing, located at one of five apartment buildings in urban Richmond, VA. Medication Management Instrument for Deficiencies in the Elderly (MedMaIDE) was used to measure MMC during individual, face-to-face interviews. Of the 107 participants, 89% were African-American with an average age of 68.5 years (±7.2), an average of 4.9 (±2.9) comorbidities and using approximately 8 (±4.1) medications on a regular basis. The mean deficit in MMC was 3 (±2.0). The most difficult skill was naming all of the medications (69.2%) followed by stating the indication (46.7%) and knowing how or when all of the medications should be taken or identifying existing refills (38.3%). Many older residents of low-income housing experience challenges in managing medications.

